# Fine mapping of a QTL for ear size on porcine chromosome 5 and identification of high mobility group AT-hook 2 (*HMGA2*) as a positional candidate gene

**DOI:** 10.1186/1297-9686-44-6

**Published:** 2012-03-15

**Authors:** Pinghua Li, Shijun Xiao, Na Wei, Zhiyan Zhang, Ruihua Huang, Yueqing Gu, Yuanmei Guo, Jun Ren, Lusheng Huang, Congying Chen

**Affiliations:** 1 Key Laboratory for Animal Biotechnology of Jiangxi Province and the Ministry of Agriculture of China, Jiangxi Agricultural University, Nanchang 330045, People's Republic of China; 2 College of Animal Science and Technology, Nanjing Agricultural University, Nanjing 210095, People's Republic of China; 3 Professional Society for Erhualian Pig Production of Jiaoxi, Changzhou 213116, People's Republic of China

## Abstract

**Background:**

Ear size and shape are distinct conformation characteristics of pig breeds. Previously, we identified a significant quantitative trait locus (QTL) influencing ear surface on pig chromosome 5 in a White Duroc × Erhualian F_2 _resource population. This QTL explained more than 17% of the phenotypic variance.

**Methods:**

Four new markers on pig chromosome 5 were genotyped across this F_2 _population. RT-PCR was performed to obtain expression profiles of different candidate genes in ear tissue. Standard association test, marker-assisted association test and F-drop test were applied to determine the effects of single nucleotide polymorphisms (SNP) on ear size. Three synthetic commercial lines were also used for the association test.

**Results:**

We refined the QTL to an 8.7-cM interval and identified three positional candidate genes i.e. *HMGA2*, *SOX5 *and *PTHLH *that are expressed in ear tissue. Seven SNP within these three candidate genes were selected and genotyped in the F_2 _population. Of the seven SNP, *HMGA2 *SNP (JF748727: g.2836 A > G) showed the strongest association with ear size in the standard association test and marker-assisted association test. With the F-drop test, F value decreased by more than 97% only when the genotypes of *HMGA2 *g.2836 A > G were included as a fixed effect. Furthermore, the significant association between g.2836 A > G and ear size was also demonstrated in the synthetic commercial Sutai pig line. The haplotype-based association test showed that the phenotypic variance explained by *HMGA2 *was similar to that explained by the QTL and at a much higher level than by *SOX5*. More interestingly, *HMGA2 *is also located within the dog orthologous chromosome region, which has been shown to be associated with ear type and size.

**Conclusions:**

*HMGA2 *was the closest gene with a potential functional effect to the QTL or marker for ear size on chromosome 5. This study will contribute to identify the causative gene and mutation underlying this QTL.

## Background

Ear size and erectness are important conformation characteristics of pig breeds. Moreover, ear defects have been observed in different species [1,2]. For example, in humans, congenital underdevelopment of the external ear or microtia, affects about 1 in 6000 children in Europe and the USA, and 1 in 4000 in Japan and China [3]. Pig can be used as an animal model to investigate the underlying mechanisms responsible for the development of the external ear. However, few genetic analyses have been carried out to study ear development in pigs and to our knowledge, only three studies have reported data on the mapping of QTL (Quantitative Trait Loci) for pig ear traits. In 2004, Guo *et al*. (in Chinese) [4] showed the existence of a QTL for ear erectness on pig chromosome 6 or SSC6 (SSC for *Sus scrofa*) in a commercial population. In 2007, Wei *et al*.[5] identified two significant QTL with a major effect on ear size on SSC5 and SSC7 in a Large White × Meishan F_2 _resource population. Another study involving a White Duroc × Erhualian F_2 _intercross also detected two major QTL for ear size on the same chromosomes, which explained more than 45% and 17% of the phenotypic variance, respectively [6]. Although the genetic positions of the QTL were different (51 cM vs. 70 cM on SSC5 and 70 cM vs. 58 cM on SSC7), the 95% confidence interval of both QTL overlapped partly in the two studies. More recently, a missense mutation on *PPARD *was identified as the causative mutation for the QTL on SSC7, which influences ear size by mediating down-regulation of β-catenin and its target gene expression [7]. Concerning the QTL on SSC5, the confidence interval was too large (11 cM) for positional cloning analysis and the genetic basis of the QTL remains unexplored.

Thus, the study reported here, aimed at investigating the causative gene for ear size on SSC5 by refining the QTL region using higher-density markers on this chromosome and at analyzing in further detail, the association between the three candidate genes located in the refined region with ear size in the White Duroc × Erhualian F_2 _resource population and three synthetic commercial lines. The results will contribute to the final characterization of the causative gene or mutation responsible for this QTL.

## Methods

### Animals and phenotypic measurements

The White Duroc × Erhualian F_2 _resource population and phenotypic measurements have been described in detail in Ma *et al*.[6]. Briefly, the F_2 _resource population was obtained by mating two White Duroc sires with small and erect ears to 17 Erhualian dams with large and floppy ears to produce F_1 _animals and then by random crossing between nine F1 boars and 59 F1 sows (avoiding sister-brother mating) to produce a total of 1912 F_2 _animals. Among the F2 individuals, 1029 were phenotyped by measuring separately the size of both left and right ears after slaughter at 240 days of age as described in our previous report [6].

Three synthetic commercial lines including Sutai (Duroc × Erhualian, n = 173), Sujiang (Duroc × Jiangquhai, n = 105) and Suzhong (Landrace × Erhualian, n = 78) were used in association tests to confirm the association between candidate genes and ear size. Both Erhualian and Jiangquhai indigenous breeds have large ears. Measurement of ear size was done according to the method described by Ren *et al*.[7], which differs from that of Ma *et al*.[6]. Briefly, the ears of each animal were fixed and photographed with a ruler placed on the surface of the ear as an internal size reference. The surface of each ear was calculated using the Leica Qwin software. All experimental animal procedures were conducted according to the guidelines for the care and use of experimental animals established by the Ministry of Agriculture of China.

### Microsatellite genotyping

Genomic DNA was extracted from ear and spleen tissues according to the routine phenol/chloroform extraction method and diluted to a final concentration of 20 ng/μL. To increase marker density in the QTL candidate region, microsatellite markers were selected from the *SWR453*-*SW1987 *interval on the USDA-MARC SSC5 linkage map [8] or by mining genomic sequence data of the QTL region (Sscrofa 9.2) using the SSRHunter program [9]. Four informative microsatellite markers, namely *SSC5P3*, *SSC5P13*, *SSC5P16 *and *SW2003*, were genotyped in all F_0_, F_1 _and 1029 F_2 _animals as described previously [10]. All the primers were designed using the online software Primer3 [11] and primer sequences, annealing temperatures, amplicon lengths and number of alleles are shown in Additional file 1: Table S1.

### Selection of candidate genes and RT-PCR

Orthologous regions between the human genome and the refined QTL region were detected using the pig-human comparative map in the pig QTL database [12]. Annotated genes present in the orthologous regions were identified with the UCSC genome browser [9] and their functions obtained from the NCBI database [13] or GeneCards [14].

*SOX5*, *HMGA2 *and *PTHLH *were selected from the refined QTL region as genes with related functions. *SOX5 *encodes a member of the SOX (SRY-related HMG-box) transcription factors family, and a previous study has indicated that it plays a role in chondrogenesis [15,16]. Moreover, mutations in the genes encoding homeobox transcription factors are responsible for the defective development of outer ears [17]. *HMGA2 *encodes a protein that belongs to the non-histone chromosomal high mobility group (HMG) protein family. *HMGA2*-*LPP *fusion protein promotes chondrogenesis by up-regulating cartilage-specific collagen gene expression through the N-terminal DNA binding domains [18]. The presence of HMGA proteins increases the proliferative activity of chondrocytes cultured *in vitro *[19]. More interestingly, *HMGA2*-deficient mice develop smaller ears [20] and in dogs, *HMGA2 *may be involved in differences in the size and type of ears [21]. *PTHLH *is a member of the parathyroid hormone family which regulates endochondral bone development through regulation of chondrocyte proliferation and differentiation during early bone growth [22]. The Wnt/beta-catenin pathway interacts differentially with the parathyroid hormone-related protein (PTHRP), which acts as a control of chondrocyte hypertrophy and final maturation [23]. Cartilage is the major component of ear tissue and genes influencing chondrocyte proliferation have been shown to affect ear size [24]. Thus, based on both the physiological roles of *SOX5*, *HMGA2 *and *PTHLH *in chondrogenesis and cartilage formation and their expression in ear tissues, we consider that they are important candidate genes for ear size.

RT-PCR was performed to check expression profiles of *HMGA2,SOX5 *and *PTHLH *in ear tissue with the β-actin gene as an internal control. Ear tissues were sampled from two Erhualian piglets at two days of age, immediately frozen in liquid nitrogen and finally conserved at −80°C. Total RNA was extracted with the Nucleospin RNA kit (QIAGEN, Hilden, Germany) following instructions in the manual. The first-strand cDNA was synthesized from 2 μg of total RNA according to the protocol of Omniscript reverse transcriptase kit (QIAGEN, Hilden, Germany). Primers were designed to amplify regions across the intron/exon boundaries to avoid genomic DNA contamination. Details on primers are in Additional file 1: Table S2. RT-PCR was performed in a 15 μL reaction volume with a touchdown PCR protocol.

### SNP identification and genotyping

Genomic DNA of four randomly selected F_1 _boars was used to identify SNP in the candidate genes. All the exons and part of the introns were amplified and sequenced with the primers listed in Additional file 1: Table S3. PCR amplification was carried out with a PE9700 thermal cycler (Applied Biosystem Inc., Drive Foster City, USA). After purification with the QIAquick Gel Extraction kit (Qiagen, Hilden, Germany), PCR products were bidirectionally sequenced on an ABI 3130 XL Genetic Analyzer (ABI, Foster City, USA). The sequences of each amplicon were analyzed with SeqMan in the DNAStar software package.

Informative SNP (i.e. with an allele frequency ≥ 25% in four boars) were chosen for further genotyping using the SNaPshot kit (Applied Biosystem Inc., Drive Foster City, USA) according to the manufacturer's protocol. Primers for PCR and SNaPshot extension assays are listed in Additional file 1: Table S4. After purification and denaturation at 95°C for 5 min, the SNaPshot reaction mixture was separated on an ABI 3130XL genetic analyzer for data collection. The SNP genotypes were recorded and analyzed using the GeneScan version 4.0.

### Statistical analysis

The linkage map of SSC5 was re-constructed by CRIMAP 2.4 [25]. The physical positions of the seven SNP on SSC5 were determined by the SOAP2 tool based on the reference genome assembly of Sscrofa 10.2 [26]. QTL analyses were carried out with QTL Express [27]. The statistical model was similar to the initial QTL mapping model [6]. Briefly, batch and sex were treated as fixed effects, and carcass weight as a covariate. The 95% confidence interval (CI) was determined using a bootstrap method through 2000 iterations [28]. A multiple QTL model was used to exclude the effects of previously detected QTL on other chromosomes by treating them as fixed effects. The significance threshold was determined with 1000 permutation tests [29].

Because of the extensive between-breed linkage disequilibrium (LD) in the F_2 _resource populations, standard association test, marker-assisted association test and F-drop test were performed to analyze the association between the candidate genes and the ear size in the current F_2 _resource population as described in Zhao *et al*.[30]. Briefly, the following models were used:

Model 1: $y_{ijkl}=\mu +batch_{i}+gender_{j}+PPARD_{k}+\beta cw_{ijkl}+e_{ijkl};$

Model 2: $y_{ijkl}=\mu +batch_{i}+gender_{j}+PPARD_{k}+\beta cw_{ijkl}+C_{a}a+C_{d}d+e_{ijkl};$

Model 3: $y_{ijkl}=\mu +batch_{i}+gender_{j}+PPARD_{k}+\beta cw_{ijkl}+CGL_{g}+e_{ijkl};$

Model 4: $y_{ijklg}=\mu +batch_{i}+gender_{j}+PPARD_{k}+\beta cw_{ijkl}+CGL_{g}+C_{a}a+C_{d}d+e_{ijkl};$

where *y_ijkl _*is the observation for ear size; *μ *is the overall mean for ear size; *batch_i _*is the fixed effect of the i^th ^batch (i = 1, 2, 3, 4, 5, 6); *gender_j _*is the fixed effect of the j^th ^sex (j = 1, 2); *PPARD_k _*is the fixed effect of the k^ith ^genotype of *PPARD *(k = 1, 2, 3); *CGL_g _*is the g^th ^genotype effect of the candidate gene (g = 1, 2, 3); *β *is the regression coefficient for carcass weight; *cw_ijkl _*is the covariate of the carcass weight; a and d are unknown additive and dominance effects of average QTL alleles by breed origin from the two F_0 _breeds, respectively, and C_a _and C_d _are the additive and dominance coefficients, as computed from marker information [31]; and *e_ijkl _*is the residual. The standard association test was based on an F ratio of residual sums of squares (RSS) for models 1 and 3. An F ratio reaching or exceeding the significant threshold means that the candidate gene is in LD with the QTL or is the QTL. The marker-assisted association test for candidate genes was based on an F ratio of RSS for models 4 and 2. In this case, if the F ratio reaches or exceeds the significant threshold, then the candidate gene locus can account for most of the phenotype variance effect and it is in LD with the quantitative trait nucleotide (QTN) or is the QTN [30]. The marker-assisted association test removed the impact of between-breed LD. The F-drop test was based on fitting the models 4 and 3. If a candidate gene that is in LD with the QTL is included as a fixed effect, it is expected to absorb part of the between-breed QTL effect, and the F-ratio for the QTL is expected to drop. All the tests were carried out with GridQTL version 2.0.0 [32].

The haplotype phases were inferred using AlphaPhase [33]. The phenotypes were corrected for sex, batch, carcass weight and additive infinitesimal effects using Qxpak 5 [34]. The residuals were regressed on the number of copies of each haplotype allele separately across all the individuals through the GLM function in R. The *P *values were corrected for the number of haplotypes (Bonferroni correction). The phenotypic variance explained by all the haplotypes was estimated by the ANOVA function in R. The association of *HMGA2 *JF748727: g.2836 A > G with ear size in three synthetic commercial lines was evaluated by the GLM procedure of SAS 9.0 (SAS® Institute Inc. Cary, NC). The stratification effect of the population was excluded in the analysis.

## Results

### Fine-mapping of the QTL

**Figure 1:**
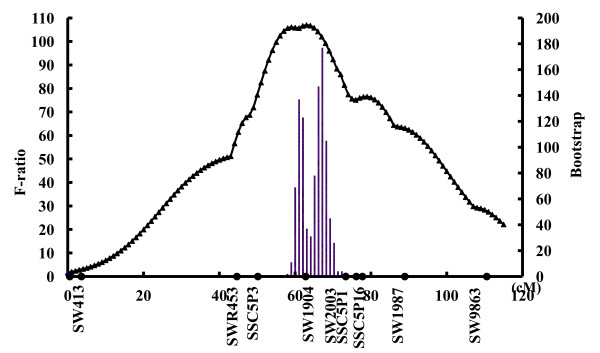
**F-value curve and bootstrap histograms of the QTL on SSC5 for ear size**. The left Y-axis indicates the F-value in QTL fine-mapping and the right Y-axis shows the number of iterations in bootstrapping.

To refine the linkage map of the region previously reported on SSC5, four additional microsatellite markers (*SSC5P3, SW2003, SSC5P13 *and *SSC5P16*) were identified and genotyped on F_0_, F_1 _and 1029 F_2 _pigs. Thus, the complete linkage map with distances between markers in cM is the following: *Acr - 3.0 - SW413 - 40.0 - SWR453 - 5.4 - SSC5P3 - 12.3 - SW1904 - 10.3 - SW2003 - 2.7 - SSC5P13 - 1.6 - SSC5P16 - 10.9 - SW1987 - 21.1 - SW986 - 7.9 - SW995*. This new round of QTL mapping reduced the confidence interval of the initial 11 cM QTL region between markers *SWR453 *and *SW1987 *to an 8.7 cM interval between markers *SSC5P3 *and *SW2003*. One thousand permutation tests were carried out and the bootstrap histograms are shown in Figure 1. The most likely position of the QTL is located in a 8.7 cM region (between positions 55.6 cM and 64.3 cM). Three genes with related functions i.e. *HMGA2*, *SOX5 *and *PTHLH *are located within this refined QTL interval. RT-PCR showed that the three genes are expressed in ear tissue (Additional file 2: Figure S1).

### Polymorphisms in candidate genes

We detected 19 SNP and four indels in the *HMGA2 *gene (accession number: JF748727, Additional file 1: Table S3). None of these polymorphisms were located in the coding region. We found 28 polymorphisms including 27 SNP and one indel in the *SOX5 *gene (accession number: JF748729, Additional file 1: Table S3) and among all these polymorphisms, only one synonymous SNP was detected in exon 2. A total of 20 SNP and one indel were identified in the *PTHLH *gene including one missense mutation in exon 2 (accession number: JF748728, Additional file 1: Table S3). Although a number of mutations were identified in the three candidate genes, most of them were identified in only one animal and thus were not informative and not useful for association tests on the F_2 _resource population. Finally, only seven SNP for which the minor allele frequency was > 25% were chosen for further genotyping across the entire White Duroc × Erhualian intercross.

### Association of candidate genes with ear size in the F_2 _resource population

The physical positions of the seven SNP on the reference genome assembly 10.2 were 32.483 Mb, 32.486 Mb, 32.497 Mb, 46.968 Mb, 51.430 Mb, 51.431 Mb and 51.480 Mb, respectively and were all situated within the interval between SSC5P3 (18.299 Mb) and SW2003 (54.594 Mb). Among the seven SNP, three included within the *HMGA2 *gene are located near the SW1904 marker (33.850 Mb), which is in the middle of the QTL. Standard association test, marker-assisted association test and F-drop test were carried out to evaluate the association between the seven informative SNP and ear size in the F_2 _resource population. In the standard association test, all the SNP were in strong association with ear size (*P*_-*sa *_< 0.01, Table 1). *HMGA2 *g.2836 A > G showed the strongest association with ear surface. Allele [G] present in Erhualian pigs is associated with an increase in ear size, while allele [A] is responsible for a smaller ear surface i.e. 32.84 ± 5.46 cm^2 ^(Table 1).

**Table 1:** *P *values obtained in standard association test and marker-assisted association test and additive effects of the seven SNP

Gene	Polymorphism	*P*_-*sa*_	*P*_-*ma*_	Allele	Additive effect ± s.e. (cm^2^)
*HMGA2 *(JF748727)	g.2836 A > G	9.19E-46	1.92E-05	A	-32.84 ± 5.46
	g.215 C > T	5.04E-26	1.23E-03	T	27.40 ± 5.37
	g.1799 G > A	1.60E-19	0.04	G	22.54 ± 4.76
*SOX5 *(JF748729)	g.9084 T > C	3.51E-07	0.03	C	32.56 ± 7.25
	g.9908 T > C	4.54E-25	0.02	C	15.72 ± 5.00
	g.12328 T > G	8.12E-07	0.11	G	-15.28 ± 5.02
*PTHLH *(JF748728)	g.9440 T > C	5.12E-07	0.03	C	11.74 ± 5.15

*P*_-*sa*_: *P *values in standard association analysis; *P*_-*ma*_: *P *values in marker-assisted association analysis; s.e.: standard error; the negative values indicate decrease in ear size

**Figure 2:**
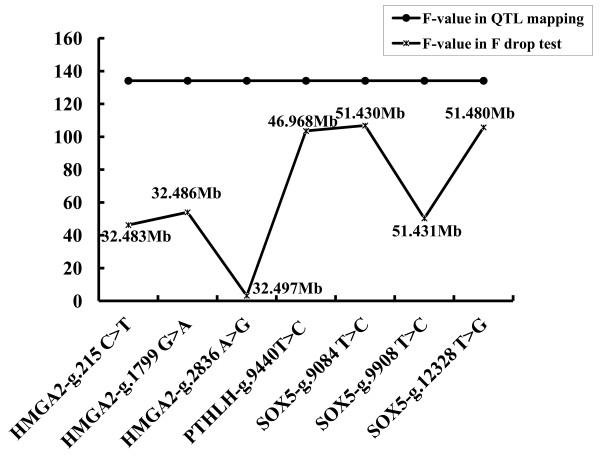
**F-value curves of seven SNP in the F-drop test when each SNP was included as a fixed effect**. The Y-axis shows F-values and the X-axis indicates the SNP order; F (QTL): F value in QTL mapping (F-values of the model 3), F (drop): F value when the candidate SNP was included as a fixed effect in QTL mapping (values of the F-ratios between models 4 and 3)

In the marker-assisted association test, apart from the SNP *SOX5 *g.12328 T > G that did not achieve a *P *< 0.05 significance level, the SNP analyzed for the three candidate genes were significantly associated with ear size (*P_-ma _*< 0.05, Table 1). Similar to the standard association test, *HMGA2 *g.2836 A > G was most strongly associated with ear size (*P_-ma _*= 1.92E-05). The results of the F-drop test are shown in Figure 2. When the genotypes of *HMGA2 *g.2836 A > G were included as a fixed effect in the QTL analysis, the F value dropped from 134.14 to 3.19. F values of the other SNP were below 90%.

*HMGA2 *and *SOX5 *haplotypes were constructed and their associations with ear size were also analyzed in this study. Overall, the phenotypic variance explained by *HMGA2 *haplotypes reached 19.47% (*P *= 1.31E-31, Table 2). Haplotypes [GCA] and [GTG] were associated with an increase in ear size. Five *SOX5 *haplotypes showed a significant association with ear size. Animals with haplotype [TCT], [TCG] or [CCT] showed larger phenotypic values than those with haplotype [TTT] or [TTG] (Table 2). However, the phenotypic variance explained by *SOX5 *haplotypes was lower than that of the QTL or *HMGA2 *haplotypes (12.82%, *P *= 2.48E-22).

**Table 2:** Associations of *HMGA2 *and *SOX5 *haplotypes with ear size in the F_2 _resource population

Gene	Haplotype	Frequency	Effect ± s.e.* (cm^2^)	Corrected *P*-value
*HMGA2*	A/C/G	0.002	-41.43 **± **28.64	1
	A/C/A	0.012	-5.09 **± **9.66	1
	A/T/G	0.484	-20.96 **± **1.70	4.34E-31
	A/T/A	0.004	-36.21 **± **15.33	0.15
	G/C/G	0.001	8.73 **± **28.68	1
	G/C/A	0.255	22.71 **± **2.22	3.87E-22
	G/T/G	0.213	16.66 **± **2.71	9.71E-09
	G/T/A	0.029	3.72 **± **6.66	1
phenotypic variance explained by all haplotypes			19.47%	1.31E-31
*SOX5*	T/T/T	0.170	-7.99 **± **2.69	0.03
	T/C/T	0.234	11.09 **± **2.33	1.83E-05
	T/T/G	0.359	-17.75 **± **1.96	6.45E-18
	T/C/G	0.101	11.85 **± **2.97	5.66E-04
	C/T/T	0.002	45.88 **± **19.90	0.17
	C/C/T	0.114	15.19 **± **2.96	2.71E-06
	C/T/G	0.008	3.87 **± **10.37	1
	C/C/G	0.012	17.88 **± **8.99	0.38
phenotypic variance explained by all haplotypes			12.82%	2.48E-22

Effect ± s.e. represent the effect of haplotypes and its standard error, the negative values indicate decrease in ear size

### Association of *HMGA*2 g.2836 A > G with ear size in outbred populations

Given that the strongest association between *HMGA2 *g.2836 A > G and ear size was identified in the White Duroc × Erhualian F_2 _resource population, this SNP was further genotyped in 356 adult pigs from the synthetic commercial lines of Sutai, Sujiang and Suzhong. The results showed that it was significantly associated with ear size in Sutai pigs (*P *< 0.05), but not in Sujiang and Suzhong pigs (Table 3) and allele [G] was responsible for an increase in ear size.

**Table 3:** Association of *HMGA2 *g.2836 A > G with ear size in synthetic commercial lines

Population	Genotype	N	Mean ± s.d.* (cm^2^)
Sutai	[AA]	90	258.57 ± 40.29^a^
	[AG]	76	292.60 ±46.28^b^
	[GG]	7	323.50 ±37.96^c^
Sujiang	[AA]	57	252.73 ±45.90
	[AG]	41	265.14 ±41.23
	[GG]	7	280.23 ±45.87
Suzhong	[AA]	47	216.61 ±38.34
	[AG]	28	224.55 ±59.25
	[GG]	3	226.68 ±80.23

*phenotypes were corrected for sex, batch and farm; the mean values marked with different superscript letter a, b and c indicate significant phenotype differences between each genotype (*P *< 0.05)

## Discussion

Identification of causative genes underlying QTL in domestic animals is still a major challenge because confidence intervals of QTL are typically too large for positional cloning of the underlying gene [35]. In this study, we refined the interval containing the QTL responsible for ear size on SSC5 from an 11 cM interval to an 8.7 cM region. However, the confidence interval of the refined QTL is still large. The linkage disequilibrium-based haplotype-sharing approach has been shown to be effective in fine mapping of QTL in domestic animals [35,36]. Unfortunately, in our study QTL genotypes could be deduced for only three of the nine F_1 _boars by the marker-assisted segregation analysis (data not shown) and no obvious shared haplotype was observed, which hampered further mapping of the QTL.

In this study, the refined QTL region corresponds to two orthologous regions on human chromosome 12 (65.70-75.07 Mb and 19.80-33.72 Mb), in which three candidate genes with related functions i.e. *SOX5*, *HMGA2 *and *PTHLH *were identified. The standard association test, marker-assisted association test and F-drop test were used to test the association between these candidate genes and ear size in the F_2 _resource population in which extensive linkage disequilibrium exists [30]. *HMGA2 *g.2836 A > G showed the strongest association with ear size both in standard association test and marker-assisted association test. Allele [G] originating from Erhualian pigs was responsible for the increase in ear size, which is consistent with the breed's characteristic large and floppy ears. With the F-drop test, F value dropped by more than 97% only if the *HMGA2 *g.2836 A > G genotype was included as a fixed effect. The phenotypic variance explained by the *HMGA2 *haplotypes was a little higher than that by the QTL (19.47% vs.17.14%). This could be due to the elimination in the association tests of the effect of the causative mutation in *PPARD *involved in the other major QTL for ear size on SSC7. However, the phenotypic variance explained by *SOX5 *haplotypes is much lower than that by the QTL (12.82% vs. 17.14%). Furthermore, the statistical models used in this study can be applied to determine which candidate gene is closest to the QTL. If *HMGA2 *is more strongly associated with the QTL than *SOX5 *and *PTHLH*, the residual sum of the squares of *HMGA2 *(RSS (*HMGA2*)) in model 3 is expected to be smaller than that of RSS (*SOX5*) and RSS (*PTHLH*) [30]. We found that the ratios of RSS (*HMGA2*)/RSS (*SOX5*) and RSS (*HMGA2*)/RSS (*PTHLH*) were < 1 (0.79 and 0.78, respectively). These results suggest that *HMGA2 *is the gene closest to the QTL.

The significant association between *HMGA2 *g.2836 A > G and ear size was confirmed in the synthetic commercial line examined in this study. The founder animals of this line and the White Duroc × Erhualian F_2 _resource population originate from the same pig breeds, i.e. Duroc and Erhualian pigs. However, no significant association was detected in the Sujiang and Suzhong pig populations, which is most likely due to: 1) the different genetic background of the populations; 2) this SNP is only in LD with the causative mutation. Because recombination exists between the SNP and the causative mutation in the Suzhong and Sujiang populations, no significant association can be detected.

More interestingly, an across-breed genome-wide association study (GWAS) in dogs showed that the SNP most strongly associated with dropping ears and ear size is located 98 kb apart from each other between the *MSRB3 *and *HMGA2 *genes [37]. In addition, an association between *HMGA2 *and ear type has been described in dogs [21] and *HMGA2*-deficient mice are known to have small ears [20]. Combined with the results obtained in this study, we suggest that *HMGA2 *is the closest gene to the QTL or the marker for ear size on SSC5 in pigs. More refined mapping of the QTL is needed to demonstrate that *HMGA2 *is responsible for ear size in pig.

## Conclusions

We have fine-mapped a QTL affecting ear size on SSC5 to an 8.7-cM region. Candidate gene analyses suggest that *HMGA2 *is the closest gene with a potential functional effect to the QTL or marker for ear size on SSC5. These results will contribute to the identification of the causative mutation for this QTL.

## Competing interests

The authors declare that they have no competing interests.

## Authors' contributions

PHL performed the experiments and wrote the manuscript; SJX collected the samples and performed the experiments; NW performed the experiments; ZYZ analyzed the data; RHH and YQG collected the samples; YMG analyzed the data; JR, provided comments and revisions for the manuscript; LSH conceived and designed the experiments and revised the manuscript; CYC performed the experiments, analyzed the data, wrote and revised the manuscript. All authors read and approved the final manuscript.

## Supplementary Material

Additional_file_ 1 **Primers for QTL fine-mapping, gene expression analyses, and SNP identification and genotyping**. **Table S1 **contains primer sequences, annealing temperatures and amplicons of the microsatellite markers, **Table S2 **primers for gene expression analyses, **Table S3 **sequences and assay conditions of the primers for SNP identification and **Table S4 **sequences and assay conditions of the primers for SNP genotyping. Click here for file

Additional_file_ 2 **The expression profiles of three candidate genes in ear tissue by RTPCR**. **Figure S1 **shows the expression level of *HMGA2*, *SOX5 *and *PTHLH *in ear tissue obtained by RT-PCR. Click here for file

## References

[B1] Djorbineva MK Aleksieva SA Lauvergne JJ Hereditary shortening of the external ear in Karakachan sheep of Bulgaria. Brief report (1) Rec Med Vet 1985 161 57 58

[B2] Bongers EM Opitz JM Fryer A Sarda P Hennekam RC Hall BD Superneau DW Harbison M Poss A van Bokhoven H Hamel BC Knoers NV Meier-Gorlin syndrome: report of eight additional cases and review Am J Med Genet 2001 102 115 124 10.1002/ajmg.1452 11477602

[B3] Aguilar EF Auricular reconstruction in congenital anomalies of the ear Facial Plast Surg Clin North Am 2001 9 159 169 11465003

[B4] Guo XL Looft C Reinsch N Ernst K QTL mapping for ear shape based on a commercial pig population Yi Chuan Xue Bao 2004 31 819 821 15481537

[B5] Wei WH de Koning DJ Penman JC Finlayson HA Archibald AL Haley CS QTL modulating ear size and erectness in pigs Anim Genet 2007 38 222 226 10.1111/j.1365-2052.2007.01591.x 17459018

[B6] Ma J Qi W Ren D Duan Y Qiao R Guo Y Yang Z Li L Milan D Ren J Huang L A genome scan for quantitative trait loci affecting three ear traits in a White Duroc × Chinese Erhualian resource population Anim Genet 2009 40 463 467 10.1111/j.1365-2052.2009.01867.x 19392826

[B7] Ren J Duan YY Qiao RM Yao F Zhang ZY Yang B Guo YM Xiao SJ Wei RX Ouyang ZX Ding NS Ai HS Huang LS A missense mutation in PPARD causes a major QTL effect on ear size in pigs PLoS Genet 2011 7 e1002043 10.1371/journal.pgen.1002043 21573137 PMC3088719

[B8] Rohrer GA Alexander LJ Hu Z Smith TP Keele JW Beattie CW A comprehensive map of the porcine genome Genome Res 1996 6 371 391 10.1101/gr.6.5.371 8743988

[B9] UCSC Pig Genome database http://genome.ucsc.edu/cgi-bin/hgGateway

[B10] Guo Y Mao H Ren J Yan X Duan Y Yang G Ren D Zhang Z Yang B Ouyang J Brenig B Haley C Huang L A linkage map of the porcine genome from a large-scale White Duroc × Erhualian resource population and evaluation of factors affecting recombination rates Anim Genet 2009 40 47 52 10.1111/j.1365-2052.2008.01802.x 19178432

[B11] Primer 3 http://frodo.wi.mit.edu/primer3/

[B12] Pig QTL database http://www.animalgenome.org/cgi-bin/QTLdb/SS/index

[B13] NCBI http://www.ncbi.nlm.nih.gov/

[B14] GeneCards http://www.genecards.org/

[B15] Lefebvre V Li P de Crombrugghe B A new long form of Sox5 (L-Sox5), Sox6 and Sox9 are coexpressed in chondrogenesis and cooperatively activate the type II collagen gene EMBO J 1998 17 5718 5733 10.1093/emboj/17.19.5718 9755172 PMC1170900

[B16] Smits P Li P Mandel J Zhang Z Deng JM Behringer RR de Crombrugghe B Lefebvre V The transcription factors L-Sox5 and Sox6 are essential for cartilage formation Dev Cell 2001 1 277 290 10.1016/S1534-5807(01)00003-X 11702786

[B17] Fekete DM Development of the vertebrate ear: insights from knockouts and mutants Trends Neurosci 1999 22 263 269 10.1016/S0166-2236(98)01366-6 10354604

[B18] Kubo T Matsui Y Goto T Yukata K Yasui N Overexpression of HMGA2-LPP fusion transcripts promotes expression of the alpha 2 type XI collagen gene Biochem Biophys Res Commun 2006 340 476 481 10.1016/j.bbrc.2005.12.042 16375854

[B19] Richter A Hauschild G Murua Escobar H Nolte I Bullerdiek J Application of highmobility-group-A proteins increases the proliferative activity of chondrocytes in vitro Tissue Eng Part A 2009 15 473 477 10.1089/ten.tea.2007.0308 18721076

[B20] Xiang X Benson KF Chada K Mini-mouse: disruption of the pygmy locus in a transgenic insertional mutant Science 1990 247 967 969 10.1126/science.2305264 2305264

[B21] Boyko AR Quignon P Li L Schoenebeck JJ Degenhardt JD Lohmueller KE Zhao K Brisbin A Parker HG vonHoldt BM Cargill M Auton A Reynolds A Elkahloun AG Castelhano M Mosher DS Sutter NB Johnson GS Novembre J Hubisz MJ Siepel A Wayne RK Bustamante CD Ostrander EA A simple genetic architecture underlies morphological variation in dogs PLoS Biol 2010 8 e1000451 10.1371/journal.pbio.1000451 20711490 PMC2919785

[B22] Tenne M McGuigan F Jansson L Gerdhem P Obrant KJ Luthman H Akesson K Genetic variation in the PTH pathway and bone phenotypes in elderly women: evaluation of PTH, PTHLH, PTHR1 and PTHR2 genes Bone 2008 42 719 727 10.1016/j.bone.2007.12.005 18280230

[B23] Guo X Mak KK Taketo MM Yang Y The Wnt/beta-catenin pathway interacts differentially with PTHrP signaling to control chondrocyte hypertrophy and final maturation PLoS One 2009 4 e6067 10.1371/journal.pone.0006067 19557172 PMC2698152

[B24] Kusuhara H Isogai N Enjo M Otani H Ikada Y Jacquet R Lowder E Landis WJ Tissue engineering a model for the human ear: assessment of size, shape, morphology, and gene expression following seeding of different chondrocytes Wound Repair Regen 2009 17 136 146 10.1111/j.1524-475X.2008.00451.x 19152661

[B25] Green P Falls K Crooks S Documentation for CRIMAP, Version 2.4 1990 Washington University School of Medicine, St Louis, MO

[B26] Li R Yu C Li Y Lam TW Yiu SM Kristiansen K Wang J SOAP2: an improved ultrafast tool for short read alignment Bioinformatics 2009 25 1966 1967 10.1093/bioinformatics/btp336 19497933

[B27] Seaton G Haley CS Knott SA Kearsey M Visscher PM QTL Express: mapping quantitative trait loci in simple and complex pedigrees Bioinformatics 2002 18 339 340 10.1093/bioinformatics/18.2.339 11847090

[B28] Visscher PM Thompson R Haley CS Confidence intervals for QTL locations using bootstrapping Genetics 1996 143 1013 1020 8725246 10.1093/genetics/143.2.1013 PMC1207319

[B29] Churchill GA Doerge RW Empirical threshold values for quantitative trait mapping Genetics 1994 138 963 971 7851788 10.1093/genetics/138.3.963 PMC1206241

[B30] Zhao H Rothschild MF Fernando RL Dekkers JC Tests of candidate genes in breed cross populations for QTL mapping in livestock Mamm Genome 2003 14 472 482 10.1007/s00335-002-2215-y 12925896

[B31] Haley CS Knott SA Elsen JM Mapping quantitative trait loci in crosses between outbred lines using least squares Genetics 1994 136 1195 1207 8005424 10.1093/genetics/136.3.1195 PMC1205874

[B32] Seaton G Hernandez J Grunchec JA White I Allen J De Koning DJ Wei W Berry D Haley C Knott S GridQTL: a grid portal for QTL mapping of compute intensive datasets Proceedings of the 8th World Congress on Genetics Applied to Livestock Production Belo Horizonte 2006

[B33] AlphaPhase http://sites.google.com/site/hickeyjohn/alphaphase

[B34] Pérez-Enciso M Misztal I Qxpak.5: old mixed model solutions for new genomics problems BMC Bioinformatics 2011 12 202 10.1186/1471-2105-12-202 21612630 PMC3123239

[B35] Georges M Mapping, fine mapping, and molecular dissection of quantitative trait loci in domestic animals Annu Rev Genomics Hum Genet 2007 8 131 162 10.1146/annurev.genom.8.080706.092408 17477823

[B36] Nezer C Collette C Moreau L Brouwers B Kim JJ Giuffra E Buys N Andersson L Georges M Haplotype sharing refines the location of an imprinted quantitative trait locus with major effect on muscle mass to a 250-kb chromosome segment containing the porcine IGF2 gene Genetics 2003 165 277 285 14504235 10.1093/genetics/165.1.277 PMC1462731

[B37] Vaysse A Ratnakumar A Derrien T Axelsson E Rosengren Pielberg G Sigurdsson S Fall T Seppälä EH Hansen MS Lawley CT Karlsson EK LUPA Consortium Bannasch D Vilà C Lohi H Galibert F Fredholm M Häggström J Hedhammar A André C Lindblad-Toh K Hitte C Webster MT Identification of genomic regions associated with phenotypic variation between dog breeds using selection mapping PLoS Genet 2011 7 e1002316 10.1371/journal.pgen.1002316 22022279 PMC3192833

