# Supplementary material for: Fine mapping of a QTL for ear size on porcine chromosome 5 and identification of high mobility group AT-hook 2 (HMGA2) as a positional candidate gene
Source: Genet Sel Evol. 2012 Mar 15;44(1):6. doi: 10.1186/1297-9686-44-6 (PMC3337325; doi:10.1186/1297-9686-44-6)
Supplement: Additional_file_ 2 — The expression profiles of three candidate genes in ear tissue by RTPCR. Figure S1 shows the expression level of HMGA2, SOX5 and PTHLH in ear tissue obtained by RT-PCR. [file 1297-9686-44-6-S2.DOC]

**Figure S1** **the expression profiles of three candidate genes in ear tissue by RT-PCR. 1: blank control; 2: β – actin; 3: *HMGA*2; 4: *PTHLH*; 5: *SOX*5; 6: 50 bp DNA ladder**

**
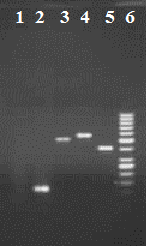
**
